# Effects of an early- and late-stage treatment with Geniposide on cognitive dysfunction in a transgenic mouse model relevant to Alzheimer's disease

**DOI:** 10.1186/1750-1326-7-S1-S4

**Published:** 2012-02-07

**Authors:** Lei Wang, Shijun Yan, Wensheng Zhang

**Affiliations:** 1 College of Resources Science & Technology, Beijing Normal University, China; 2 State Key Laboratory of Earth Surface Processes and Resource Ecology, Beijing Normal University, China; 3 Beijing Area Major Laboratory of Protection and Utilization of Tradition Chinese Medicine, Beijing Normal University, China; 4 Center for Natural Medicine Engineering, Ministry of Education of China, China

## Background

Alzheimer’s disease (AD) is an age-related, progressive neurodegenerative disorder that occurs gradually and results in memory, behavior and personality change. Geniposide, an iridoid glycosides isolated from the Traditional Chinese Medicine *Gardenia jasminoides.* Ellis, has been demonstrated to have neuroprotective effects.

## Method

The present report primarily studied the effects of geniposide on cognitive dysfunction in a double-transgenic AD mouse model (APP/PS1) on early and late stages. Male APP/PS1 mice aged 6 month and 12 month were given water, geniposide (25mg.kg^-1^) once a day by oral gavage, and age-matched C57BL/6 mice were treated with water and geniposide (25mg.kg^-1^). Aricept (0.75mg.kg^-1^) was used in the positive control group. Morris water maze performance was assessed after 3 months of treatment.

## Result

The APP/PS1 mice had longer escape latency and lower percentage of activities in platform quadrant than the C57BL/6 mice (p<0.05) both in the early- and late-stage treatment. In addition, geniposide significantly improved learning deficits as well as Aricept, compared with vehicle control treatment (p<0.05).

## Conclusion

Geniposide can improve cognitive function of the double transgenic mouse model at early and late-stage of Alzheimer’s disease, with a possible mechanism related with neuroprotective and anti-inflammatory activities. The study suggested that oral supplementation of geniposide might be a potential therapeutic strategy for the treatment of early- and late-stage of Alzheimer’s disease.

